# ACCESSING AND UTILIZING SWAN DATA AND BIOSPECIMENS FOR NEW RESEARCH QUESTIONS

**DOI:** 10.1093/geroni/igad104.2034

**Published:** 2023-12-21

**Authors:** Samar El Khoudary, Nanette Santoro, Sioban Harlow, Alicia Colvin, Nancy Avis, Carrie Karvonen-Gutierrez, Maria Brooks

**Affiliations:** University of Pittsburgh, Pittsburgh, Pennsylvania, United States; University of Colorado, Aurora, Colorado, United States; University of Michigan School of Public Health, Ann Arbor, Michigan, United States; University of Pittsburgh, Pittsburgh, Pennsylvania, United States; Wake Forest University School of Medicine, Winston-Salem, North Carolina, United States; University of Colorado, Aurora, Colorado, United States; University of Pittsburgh, Pittsburgh, Pennsylvania, United States

## Abstract

Sharing scientific resources is a critical component of contemporary research. Data and specimens from established studies can be used for manuscripts on special topics and research projects from experienced investigators and advanced trainees. Researchers external to a study, however, may find it challenging to identify available resources and to determine the steps required to access them. This presentation will describe the SWAN datasets, specimens and documents that are housed at the NIA Aging Research Biobank. It will review the process for requesting the archived resources, provide tips for successful applications, and explain the data and/or material transfer agreements created for research projects. The SWAN longitudinal cohort study aims to understand variations in the experience of reproductive aging and its impact on midlife and early old age outcomes across multiple racial and ethnic groups. SWAN includes 3302 women aged 42-54 years from 7 sites in the United States and consists of a baseline and 17 follow-up visits. The study has repeated measures of reproductive hormones, menopausal symptoms, sleep, genitourinary and sexual function, physical and cognitive function, cardiometabolic and bone health, psychological well-being and quality of life. Serum, plasma, and urine specimens were collected at each clinic visit, and a substudy obtained daily urine for ≤50 days annually for ≤10 years. An expansive collection of SWAN biospecimens and datasets are available to external investigators and trainees. Understanding the existing resources and processes required to access them provides researchers the opportunity to use these valuable resources to advance academic research.

